# Case report: long-term sustained remission in a case of metastatic colon cancer with high microsatellite instability and KRAS exon 2 p.G12D mutation treated with fruquintinib after local radiotherapy: a case report and literature review

**DOI:** 10.3389/fphar.2023.1207369

**Published:** 2023-06-28

**Authors:** Ruiqi Wang, Dan Cong, Yuansong Bai, Wenlong Zhang

**Affiliations:** Department of Hematology and Oncology, China-Japan Union Hospital of Jilin University, Changchun, China

**Keywords:** metastatic colon cancer, microsatellite instability, KRAS exon 2 p.G12D mutation, fruquintinib, radiotherapy

## Abstract

To demonstrate the efficacy of fruquintinib administration after local radiotherapy in a patient with metastatic colon cancer with high microsatellite instability and the KRAS exon 2 p. G12D mutation. The patient was administered four cycles of pembrolizumab intravenous infusion and achieved stable disease as the best outcome. He was then underwent follow-up concurrent radiochemical therapy (local DT4600cGy/23f/32d radiotherapy, and S-1 to increase sensitivity to radiotherapy), but this had little efficacy. Following this, he was administered fruquintinib and achieved sustained partial remission. At the time of last follow-up, the patient was in continuous remission for 30 months. Administration of fruquintinib after local radiotherapy may be an effective treatment for specific populations with metastatic colorectal cancer.

## Introduction

Colorectal cancer (CRC) is the third leading cause of cancer-related death in the United States (Siegel et al., 2021). Approximately 20% of patients have metastatic disease at the time of diagnosis, and 40% experience recurrence after initial treatment (Biller and Schrag, 2021). Metastatic CRC (mCRC) remains incurable. The outcome of these patients is often dismal, with a median overall survival (OS) of 15.4 months and a 5-year survival rate of <20% (Goldstein et al., 2014). Systemic treatments, including chemotherapy, antiangiogenic drugs, immune checkpoint inhibitors (ICIs), local treatment, and their combinations, are recommended for mCRC (Benson et al., 2021). Although first-line and second-line treatments can delay disease progression, most patients require third-line treatment (Marshall, 2021). Strategies to improve the survival benefits of such patients by 3 months or longer remain limited (Grothey et al., 2013; Mayer et al., 2015; Li et al., 2018). Here, we present a case of recurrent colon cancer with high microsatellite instability (MSI-H) and the KRAS G12D mutation presenting as a large abdominal mass. The patient did not respond to ICIs but has been in continuous remission for 30 months after receiving third-line sequential treatment with local radiotherapy and fruquintinib.

## Case presentation

A 53-year-old man diagnosed with sigmoid colon cancer 1 year ago underwent radical resection for colon cancer. He presented with abdominal pain and difficulty in constipation and was admitted to the hospital on December 2018. Preoperative abdominal CT revealed space occupying lesions from the sigmoid colon to the lower part of the descending colon, with multiple peripheral lymph node enlargement. Tumor markers (including carcinoembryonic antigen, carbohydrate antigen 199) were within the normal range. Due to the lack of standardized experimental procedures, circulating tumor DNA (ctDNA) analysis is generally not carried out routinely at our hospital. The postoperative pathological report showed invasion of the gastric serosa with a moderately differentiated adenocarcinoma and cancer cell infiltration of vessels without invasion. Peri-intestinal lymph node metastasis (2/12) near the lesion in the sigmoid colon and a cancer nodule were also detected. His postoperative pathological stage was pT4aN2a.m.0 IIIC. Immunohistochemical markers were as follows: Ki-67 (+70%), MLH1 (+80%), PMS2 (+80%), MSH2 (−), MSH6 (−), and p53 (+70%), indicating the MSI-H type. His relatives did not have lynch syndrome-associated cancer. Two weeks after the operation, the patient received standard adjuvant chemotherapy of sufficient duration and dosage. Eight cycles of CAPOX chemotherapy (oxaliplatin 130 mg/m^2^ was administered on day 1 and capecitabine was administered orally at a dose of 2000 mg/m^2^/day, divided into two split daily doses for 14 days followed by 7 days of rest) were administered. The patient tolerated chemotherapy well and only exhibited grade 2 hand foot syndrome. After chemotherapy, he was followed-up regularly every 3 months.

**FIGURE 1:**
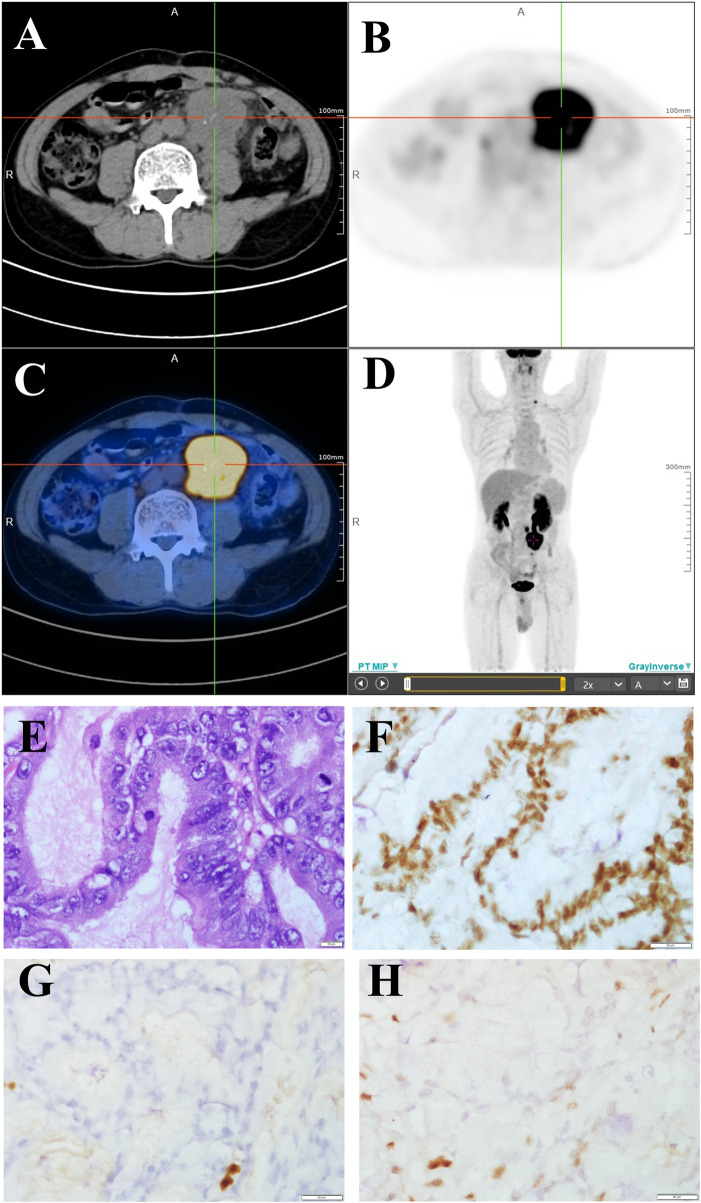
FDG PET/CT and pathological changes **(A–C)** PET/CT cross sectional view. A mass shadow with increased glucose metabolism can be seen near the anastomotic site. Its maximum SUV value is 18.18 and its size is about 49.5 × 42.5 mm. It is nodular with strip-shaped calcification and the boundary with the left ureter is unclear. **(D)** 18F-FDG PET/CT maximum density projection (MIP). Hypermetabolic nodules are visible in the left neck and lumpy lesions with increased glucose metabolism are visible in the abdomen. **(E)** Moderately differentiated adenocarcinoma (H&E staining, 400×). **(F–H)** Immunohistochemical staining showed that cells were positive for MLH1 **(F)** and negative for MSH2 **(G)** and MSH6 **(H)** (400×).

In December 2019, he developed abdominal pain and constipation again. F-fluorodeoxyglucose positron emission tomography computed tomography detected a large mass with elevated glucose metabolism near the anastomosis (Figures 1A–D), as well as multiple retroperitoneal lymph node metastases. The mass was diagnosed as moderately differentiated adenocarcinoma by biopsy pathology and was considered to originate from the intestine (Figure 1E). Immunohistochemical staining showed that cells were positive for MLH1 (Figure 1F) and negative for MSH2 (Figure 1G) and MSH6 (Figure 1H). CEA and CA 19–9 were within the normal reference value range. Next-generation sequencing (NGS) of the recurrence lesion was performed to detect clinically relevant KRAS, TP53, PIK3CA, BRAF, NRAS, FGFR1, FGFR3, FGFR2 and HER-2 mutations. Only KRAS exon 2 p. G12D mutation was detected, and the mutation allele frequency was 32.21%. The MSI score of the tumor tissue was 0.6087 (cutoff value, 0.2).

**FIGURE 2:**
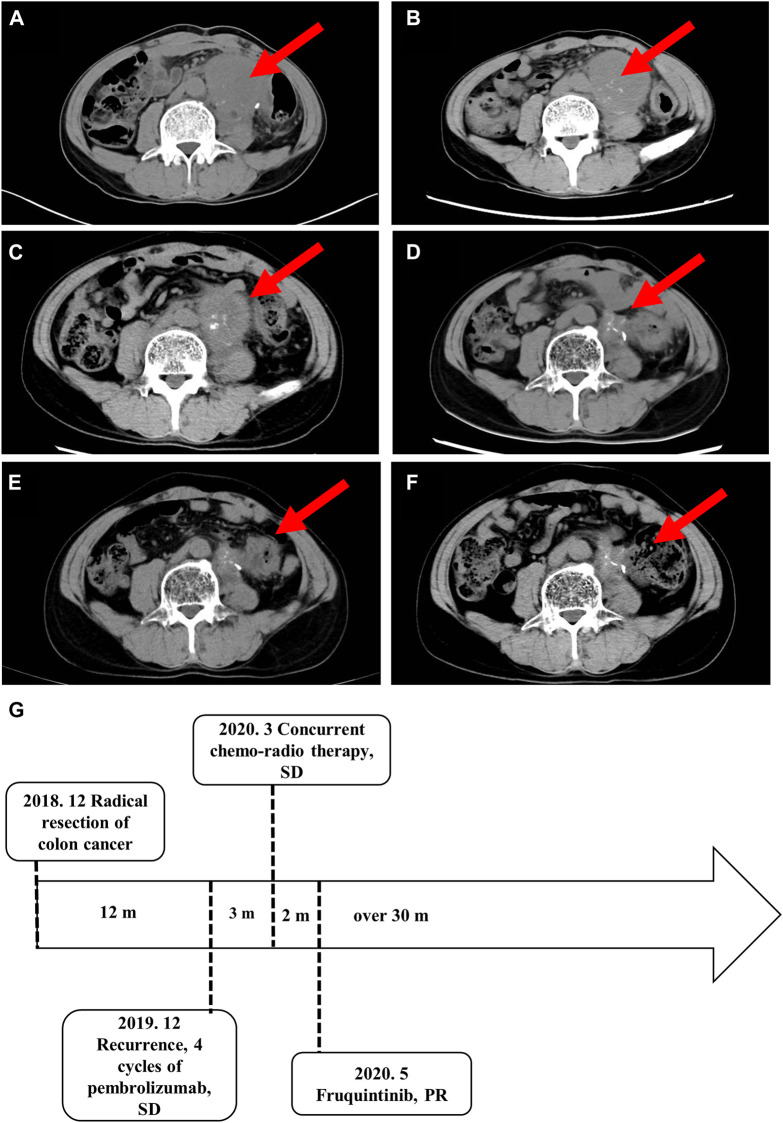
Imaging studies and the treatment time axis. Follow-up imaging of the abdominal mass. **(A)** After four cycles of pembrolizumab. **(B)** After local DT4600cGy/23f/32d radiotherapy. **(C)** Three months **(D)** 12 months **(E)**18 months, and **(F)** 30 months after the start of fruquintinib administration. **(G)** The treatment time axis. SD, stable disease; PR, partial response.

Abdominal metastatic colon cancer with MSI-H was confirmed and considered unresectable after surgical consultation. The patient received four cycles of pembrolizumab (200 mg every 3 weeks by intravenous infusion). However, the patient experienced a grade III gastrointestinal reaction after 15 weeks of immunotherapy, which manifested as loss of appetite, fatigue, and repeated diarrhea, and refused further immunotherapy. The patient refused colonoscopy for the detection of immune related enteritis. The response to treatment, as evaluated by imaging, was stable disease (Figure 2A). Because of the risk of intestinal obstruction and the ineligibility for surgical resection, the patient received local DT4600cGy/23f/32d radiotherapy. S-1, an oral fluoropyrimidine, was administered at 60 mg/m^2^ per day, orally, on days 1–14 and 29–42 in combination with radiotherapy to increase the sensitivity to radiotherapy. After radiotherapy, the abdominal pain was slightly relieved, and no serious treatment-related adverse events were observed. However, imaging follow-up showed no changes in the abdominal mass (Figure 2B). Considering that the previous efficacy was not ideal, the imaging revealed incomplete intestinal obstruction, and the patient’s general state was poor, we decided not to proceed with chemotherapy. The patient was then treated with the third-line drug fruquintinib, a small molecule multi-target tyrosine kinase inhibitor, at 5 mg orally for a 28-day treatment cycle of 3 weeks followed by 1 week off starting in May 2020.

The abdominal pain and defecation difficulties improved significantly after treatment. The abdominal mass showed continuous shrinkage in the imaging follow-up starting on the third month of fruquintinib treatment (Figures 2C–E). After 14 months of oral administration of fruquintinib, serum creatinine increased to 110.2 μmol/L and the 24-h urinary protein was 4.8 g. Therefore, the fruquintinib treatment was interrupted for 2 weeks. After renal function and urinary protein returned to normal, oral administration of fruquintinib was restarted at a reduced dose of 3 mg per day. The last imaging follow-up was at 30 months, and the focus remains obtaining a continuous partial response, as determined using the RECIST1.1 guidelines (Figures 2F, G).

## Discussion

DNA mismatch repair deficiency (MMR) was considered a biomarker of poor prognosis in the era before the advent of immune checkpoint inhibitors. MMR is present in 3%–5% of patients with metastatic colorectal cancer and is associated with tumors with a high tumor mutation burden (TMB) and MSI (Koopman et al., 2009; Venderbosch et al., 2014). The median progression free survival (PFS) and OS are significantly worse in patients with deficient DNA mismatch repair (dMMR) than in those with proficient MMR (pMMR) tumors (PFS: 6.2 vs 7.6 months, *p* = 0.001; OS: 13.6 vs 16.8 months, *p* = 0.001). The large proportion of mutant neoantigens in dMMR cancers make them sensitive to immune checkpoint blockade (Table 1). ICIs, namely, pembrolizumab or nivolumab, have shown durable antitumor activity and few treatment-related adverse events compared with chemotherapy in patients with MSI-H or dMMR mCRC in recent clinical trials (Overman et al., 2017; Diaz et al., 2022). This resulted in the US Food and Drug Administration approval of these agents for this patient population (Smith and Desai, 2018; Marcus et al., 2019). The objective remission rate (ORR) in CRC patients with MSI-H is 35.8%–45% (Overman et al., 2017; Diaz et al., 2022), indicating that more than half of the cases are at risk of stable or even disease progression. The present case did not benefit from programmed death-1 (PD-1) blocking therapy, which may be attributed to tumor intrinsic and extrinsic factors (Schreiber et al., 2011). Firstly, tumor immunity is a double-edged sword. After cancer immunoediting, existing tumor cells survive through immune escape. Secondly, the immunosuppressive state of the tumor microenvironment (TME) may provide a shelter for tumor growth. In addition, severe gastrointestinal reactions and diarrhea may cause an imbalance of the gastrointestinal flora, trigger negative immune regulation mechanisms, and could result in interruption of treatment, leading to loss of response to ICIs (Pitt et al., 2016). These factors may explain why this patient did not benefit from pembrolizumab. Another consideration is that the patient also had the KRAS exon 2 p. G12D mutation.

**TABLE 1:** Clinical trials of immuno-checkpoint inhibitors for DNA mismatch repair-deficient or microsatellite instability-high tumors.

Clinical trials	Primary site	ORR (%)	DCR (%)	CRR (%)	PFS	OS	Immuno-checkpoint inhibitors	References
KEYNOTE-177	Colorectal cancer				16.5 vs 8.2 months	NR vs 36.7 months	Pembrolizumab	Diaz et al. (2022)
KEYNOTE-158	Endometrial cancer	48			13.1 months	NR	Pembrolizumab	O'Malley et al. (2022)
CheckMate 142	Colorectal cancer	65	81	13	NR	NR	Nivolumab plus low-dose ipilimumab	Lenz et al. (2022)
GARNET-a	Endometrial cancer	43.5					Dostarlimab	Oaknin et al. (2022)
KEYNOTE-164	Colorectal cancer	33			4.1 months	NR	Pembrolizumab	Le et al. (2020)
NCT03435107	Colorectal cancer	42.4			12 months was 58.2%	12 months was 68.3%	Durvalumab	Oh et al. (2022)
NCT03150706	Colorectal cancer	28.6			8.1 months	NR	Avelumab	Kim et al. (2020)
NCT03941574	MSI-H solid tumours	38.2					Serplulimab	Qin et al. (2022)
ChiCTR-OIC-17013249	MSI-H solid tumours	66.7	100		12 months was 83.3%	12 months was 90%	Camrelizumab	Chen et al. (2020)
NCT02925234	MSI-H solid tumours	27	50		5 months	14 months	Durvalumab	Geurts et al. (2023)

ORR, objective response rate; DCR, disease control rate; CRR, complete remission rate; PFS, progression-free survival; OS, overall survival; NR, not reached.

KRAS exon 2 mutations are detected in 40% of patients with CRC (Berlin, 2013). KRAS exon 2 p. G12D mutation plays a critical role in the conversion of regulatory T cells (Tregs) and promotes an immunosuppressive TME (Table 2) (Cheng et al., 2019; Liao et al., 2019). The KRAS G12D mutation decreases the TMB, downregulates programmed death receptor ligand-1 (PD-L1), and reduces immune cell infiltration (Gao et al., 2020). Therefore, the benefit from immunotherapy may be reduced in patients with KRAS mutations. Subgroup analysis in the KEYNOTE-177 trial showed that in KRAS mutated mCRC patients with MSI-H, the survival benefit of pembrolizumab was not superior to that of chemotherapy (Diaz et al., 2022). This may explain the loss of response to ICIs in the present case.

**TABLE 2:** The effect of the KRAS-G12D mutation on the efficacy of immunotherapy.

Primary tumor	Affected cells	Secretion of cytokines	Ligand expression	Signaling pathway	TMB	Regulatory effect	References
NSCLC	CD8^+^ TILs ↓	CXCL10/CXCL11 ↓	PD-L1 ↓	P70S6K/PI3K/AKT		Negative	Liu et al. (2022)
NSCLC	CD4 memory T cell ↓, helper T cell ↓, M1 macrophage and NK cell ↓		PD-L1 ↓		Low	Negative	Gao et al. (2020)
Pancreatic cancer	Treg ↑	IL-10 ↑, TGF-β ↑				Negative	Cheng et al. (2019)
PDAC	Cancer associated fibroblasts ↑, CD8^+^ TILs ↓					Negative	Mahadevan et al. (2023)
Colorectal Cancer		IRF2 ↓, CXCL3 ↑, CXCR2 ↑				Negative	Liao et al. (2019)
Breast cancer			PD-L1 ↓	RAS/MAPK, RAS/PI3K		Negative	Liang et al. (2021)
Colorectal Cancer	CD8^+^ TILs ↓			NF-κB ↓		Negative	Liu et al. (2023)

TMB, tumor mutation burden; PD-L1, programmed death receptor ligand-1; Treg, regulatory T cells; IL-10, interleukin-10; TGF-β, transforming growth factor-β; CD8^+^ TILs, CD8^+^ tumor-infiltrating lymphocytes; PDAC, pancreatic ductal adenocarcinoma; IRF2, interferon regulatory factor 2; CXCL, chemokine (C-X-C motif) ligand; CXCR2, chemokine receptor 2.

In the present case, because the mass in the abdominal cavity was large and could not be removed surgically, local radiotherapy was selected as the second-line treatment. Radiotherapy can regulate the TME via multiple mechanisms, including promoting immunogenic cell death through the “*in situ*” vaccination effect to increase immune cell trafficking and fighting against immune evasion mechanisms orchestrated by stromal cells (Menon et al., 2019). On the other hand, radiotherapy-induced tumor tissue hypoxia promotes the release of vascular endothelial growth factor receptor 2 (VEGFR2) and tumor neovascularization (Jarosz-Biej et al., 2019). This is the theoretical basis behind the combination of small molecule anti-vascular targeted drugs with radiotherapy. A prior study showed that regorafenib increases the anti-CRC efficacy of radiotherapy by inducing apoptosis and decreasing nuclear factor kappa B signaling (Liu et al., 2020).

Third-line treatment options for metastatic colorectal cancer are still confusing. Clinical research data have shown that the survival benefit of third-line treatment is still limited. Treatment plans need to weigh potential efficacy, treatment-related adverse events, patient tolerance, and subjective acceptance. In our case, our first choice was to use ICIs. However, the patient experienced disease progression during the previous four cycles of pembrolizumab administration. The disease progression revealed by imaging follow-up may be because the treatment course was not sufficient or lymphocyte infiltration caused local edema of the tumor focus. According to the data of clinical trial KEYNOTE-177, patients with MSI-H/dMMR mCRCs who received pembrolizumab do not show significant survival benefits in the initial 6 months. After 6 months of treatment, the ICIs continued to be effective with a significant upward trend in survival benefit. This study indicates that ICIs may have a slow but long-lasting effect. Unfortunately, our case developed a degree III gastrointestinal reaction that manifested as a loss of appetite, fatigue, and repeated diarrhea. The above clinical manifestations do not exclude immune related adverse events (IRAEs). Previous studies have shown that ICIs induce diarrhea and colitis, which should be identified and treated as soon as possible; otherwise, septic shock or intestinal perforation, or even death can ensue. Therefore, rechallenge with ICIs should only proceed after weighing the clinical benefits and the risks of this treatment.

Secondly, targeting the KRAS G12D mutation seems to be an ideal choice. KRAS G12D is an important mutation in a variety of cancers but is also a challenging target. Unfortunately, most drugs targeting KRAS G12C are not effective in patients with KRAS G12D. Preclinical trials of MRTX1133, a noncovalent and highly selective G12D inhibitor, are under way. MRTX1133 treatment might have been a very promising option in a later line of treatment for our patient.

Thirdly, is not clear whether it is worth attempting chemotherapy in combination with VEGFR antibody or tyrosine kinase inhibitors (TKIs). It is worth noting that during first-line and second-line treatment, our patient was emaciated, exhibited poor nutritional status, and had a very low physical strength score. More importantly, he did not want to receive chemotherapy and wanted a chemotherapy-free regimen instead. So far, TKIs appear to be one of the few therapeutic options.

Angiogenesis plays a vital role in the development of mCRC (Hanahan and Weinberg, 2000). Fruquintinib is a small molecule inhibitor with high selectivity against VEGFR-1, -2, and -3, and it inhibits VEGF-induced phosphorylation, endothelial cell proliferation, and tubule formation (Sun et al., 2014; Deng and Li, 2019). It was first approved in China in 2018 for patients with recurrent and metastatic CRC experiencing failure of more than two lines of systematic treatment (Shirley, 2018). However, the benefits of third-line and later line therapies for mCRCs remain limited. The FRESCO study showed that, compared with placebo, fruquintinib only prolonged median OS and PFS by 2.73 and 1.87 months, respectively (Li et al., 2018).

In the present case, the PFS after treatment with fruquintinib was >30 months, which is considerably longer than that reported in clinical trials and longer than we expected. A patient with KRAS codon 12 mutation and oligometastatic CRC received radiotherapy (RT) combined with regorafenib and achieved PFS for up to 36 months (Roberto et al., 2017). Presumably, RT and anti-vascular targeted drugs may have synergistic antitumor effects. KRAS exon 2 p. G12D mutation seems to play a key role in suppressing the tumor’s response to immunotherapy. Multi-target anti-vascular tyrosine kinase inhibitors may inhibit tumor angiogenesis and improve the TME by blocking the Raf/MEK/ERK signaling pathway downstream of KRAS (Wang et al., 2015; Li et al., 2021). Only a few studies have reported using sequentially administered TKIs and RT or TKIs combined with RT. A clinical trial named REGINA aims to evaluate the survival benefits of neoadjuvant regorafenib in combination with nivolumab and short-course RT for stage II and III rectal cancer, the results of which are highly anticipated (Bregni et al., 2021). A previous case showed that a patient with metastatic hepatocellular carcinoma achieved long-term survival of over 71 months after receiving a combination of regorafenib and RT (Kim et al., 2023). RT may improve the tumor microenvironment, promote the release of tumor antigen, and synergistically increase the effects of TKIs and ICIs. This may explain the long-term sustained remission achieved in the present case. In the future, we intend to initiate clinical trials of combinations of RT and TKIs for the treatment of metastatic colorectal cancer.

In conclusion, despite a high degree of MSI, KRAS exon 2 p. G12D mutation played a critical role in the lack of response to anti-PD-1 immunotherapy in the present case. Sequential administration of local RT and fruquitinib may be an ideal treatment modality for patients with KRAS exon 2 p. G12D oligometastatic CRC. In future studies, we will establish relevant animal models to explore the synergistic mechanism and optimal administration mode of small molecule tyrosine kinase inhibitors with other therapies.

## Data Availability

The original contributions presented in the study are included in the article/Supplementary material, further inquiries can be directed to the corresponding author.
